# Long-term outcomes of distal locking in extracapsular fractures treated with trochanteric Gamma3 nails

**DOI:** 10.1186/s10195-021-00609-4

**Published:** 2021-11-26

**Authors:** Carlos Hernández-Pascual, José Ángel Santos-Sánchez, Juan Manuel García-González, Carlos Fernando Silva-Viamonte, Carmen Pablos-Hernández, Luis Ramos-Pascua, José Antonio Mirón-Canelo

**Affiliations:** 1 Department of Trauma and Orthopaedic Surgery, Hospital Universitario de Salamanca, Pso. San Vicente 58-182, 37004 Salamanca, Spain; 2 Department of Radiology, Hospital Universitario de Salamanca, Pso. San Vicente 58-182, 37004 Salamanca, Spain; 3 Department of Sociology, Universidad Pablo de Olavide, Crta. de Utrera Km. 1, 41013 Sevilla, Spain; 4 Department of Statistics, Faculty of Medicine, Universidad de Salamanca, Campus Miguel de Unamuno, Avda. Alfonso X el Sabio s/n, 37007 Salamanca, Spain; 5 Department of Geriatrics, Hospital Universitario de Salamanca, Pso. San Vicente 58-182, 37004 Salamanca, Spain; 6 Department of Trauma and Orthopaedic Surgery, Hospital Universitario 12 de Octubre, Avda. de Córdoba, s/n, 28041 Madrid, Spain; 7 Department of Preventive Medicine and Public Health, Faculty of Medicine, Universidad de Salamanca, Campus Miguel de Unamuno, Avda. Alfonso X el Sabio s/n, 37007 Salamanca, Spain

**Keywords:** Intertrochanteric fracture, Gamma3, Distal locking, Consolidation, Mechanical complications, Cut-out, Risk factor

## Abstract

**Background:**

Few publications have assessed long-term results of distal locking of short endomedullary nails for extracapsular hip fracture. Virtually all of them focus on immediate differences. Criteria for the use of static or dynamic locking are unclear in most nailing systems, and use is advised in unstable fracture patterns or with risk of bell-clapper effect, but often influenced by the “orthopaedic school”.

**Materials and methods:**

This is a historical cohort study on patients diagnosed and operated in 2014 and followed up until endpoint, considered as consolidation or major complication, plus evaluation of overall long-term survival. They were categorised as static distal locking (ST) or dynamic distal locking (DN). Both are comparable, except for all stable pre-operative classifications, Fracture Mobility Score (FMS) at discharge, and immediate post-operative loading, all of which were in favour of DN.

**Results:**

Consolidation took place in > 95% of patients, with a non-statistically significant delay trend in ST. Less than 6% in both ST and DN had major complications, with no differences. Most cases suffered early cut-out. Significant fracture collapse was the most frequent minor complication. There were more statistically significant minor and total complications in ST. Infection, without differences, can precede cut-out. Lateral thigh pain was similar and could be related to back-out. In DN, 21.1% of cases were truly dynamised. We did not find differences in mobility or in long-term survival.

**Conclusions:**

Any type of distal locking seems to be safe for consolidation, despite a slightly longer consolidation time in static locking. Early cut-out was the main complication, while others were very infrequent, which is an advantage over helical blade devices. There was a higher rate of minor and overall mechanical complications in ST, but infection and lateral thigh pain were similar. Most non-traumatic mechanical complications occurred around 5–6 weeks. About one in five of the DN truly dynamised, with all cases occurring before 8 weeks. Mobility until endpoint and overall long-term survival were not influenced by the locking mode used.

**Level of evidence:**

Therapeutic study, level 2b.

## Background

Osteoporotic hip fractures occur mainly in elderly people, and they carry a high mortality, up to 50% in patients with marked comorbidity [1]. In Spain, they are one of the main causes of admission and hospital stays [26]. The ageing of the population has turned this issue into a problem for the public health systems in developed countries [30], and it even led to the development of orthogeriatrics [48]. The treatment of extracapsular fractures (ECF) of the proximal femur is under universal consensus with strong evidence for management. They occur in cancellous, well-vascularised bone, with low risk of non-union; therefore, their treatment consists of reduction and osteosynthesis, reserving conservative treatment for patients unfit for anaesthesia [48]. Internal reduction–fixation is the treatment of choice over arthroplasty [10, 32]. An ideal implant must be easy to manage and allow complete immediate post-operative loading by sufficient fragment fixation. Dynamic Hip Screw (DHS) is the gold standard in ECF deemed as stable, whereas cephalomedullary nails are preferentially used in unstable ones [34]. Nevertheless, ease of use, familiarity technique, shorter surgery time and difficulty to define intra-operative stability has recently encouraged many surgeons to use intramedullary nails for all, though not without some controversy [53, 57].

Varus malreduction [39], ECF extension to the femoral neck [13], posterior subtype of Ikuta’s classification [8], vertical shear fractures [21] and intra-operative breakage/lack of lateral wall competency [59] have recently been added to classic instability elements of ECF: insufficient posteromedial cortical contact, avulsion of lesser trochanter, subtrochanteric extension, and reverse fracture line [6, 63]. In classic basicervical or “basicervical-equivalent” ECF, inter-fragmentary rotation is considered, so an anti-rotation device before definitive fixation is widely accepted [57]. Subtrochanteric extension of ECF determines the selection of a short or long nail, without consensus on ideal nail length [7]. In short nails, type of distal locking has not been considered in patient’s safety. Few publications, most of them retrospective and with low statistical power, can be found using long nails [63], long/short nails mixed indiscriminately [6], one single locking mode, or excluding some types of fractures, such as AO Foundation/Orthopaedic Trauma Association (AO/OTA) type A3 ones [39]. Some studies suggest dynamic locking implies higher complications [13], unlike those which report that the static approach causes further shortening, with subsequent risk of Trendelenburg gait [8, 21]. Most surgical techniques indicate them if there is risk of bell-clapper effect and in unstable ones, but it is necessary to differentiate pre- and intra-operative stability, so in the end, it is up to the surgeon in charge or the “orthopaedic school” he/she was trained in [59, 63].

Our overall goal is to find out whether the locking mode, using an internationally recognised short nail with a single cephalic screw, has any role in consolidation, in the non-traumatic mechanical complications (NTMC), or in infection and lateral thigh pain in ECF. Our specific objectives are to detect dynamisation and possible influence on mobility and overall long-term survival. Our null hypothesis is that the type of distal locking does not influence fracture consolidation, NTMC, infection or lateral thigh pain, as well as mobility or long-term survival.

## Materials and methods

This was a historical cohort study of patients exposed to surgery for extracapsular hip fractures diagnosed in our Orthopaedic Surgery and Traumatology unit; patients were older than 65 years old in 2014. The clinical research work was done at Hospital Universitario de Salamanca, a third-level university hospital and regional reference centre. This study was approved by the hospital’s research ethics committee. At least one of the following characteristics had to be met as an endpoint:

(a) Consolidation: absence of groin pain and trabeculae pass in fracture fragments, greater than 50% in both radiological projections.

(b) Major mechanical complication: one which, at least potentially, requires a new surgical intervention on the affected hip for its resolution.

**Fig. 1:**
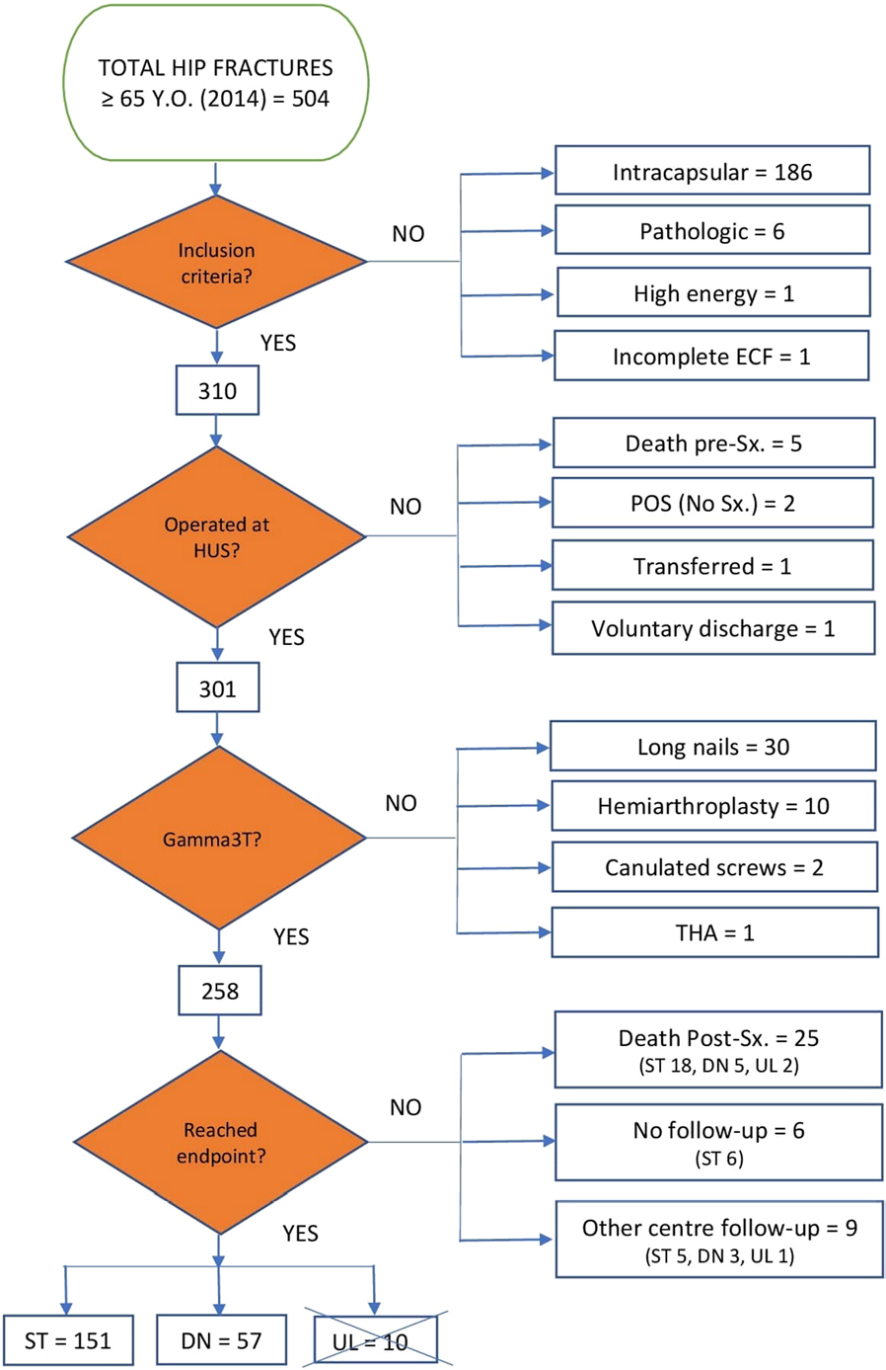
Organisation chart. ECF, extracapsular fracture; HUS, Hospital Universitario de Salamanca; Sx., surgery; POS, poor overall status; THA, total hip arthroplasty; ST, static locking mode; DN, dynamic locking mode; UL, unlocked mode

The organigram in Fig. 1 outlines the sample collection method, with inclusion and exclusion criteria. The study was carried out with 208 titanium Gamma3 trochanteric nails (hereafter referred to as Gamma3T) – length 180 mm and distal width 11 mm, neck–shaft angle between 120° and 130°, by Stryker Trauma GmbH, Schörnkirchen, Germany; a total of 151 in static locking mode (referred to as ST) and 57 in dynamic locking mode (referred to as DN). Due to their scarce representativeness and the difficulty to interpret dynamisation, the ten unlocked (UL) cases were discarded (Fig. 1).

All cases occurred in different people, except one that needed bilateral ST surgery (3 months passed between both interventions, but an endpoint criterion was met in each). All were performed by close reduction, except for two (0.96%), which needed open reduction and cerclage wiring. No anti-rotation implant was used apart from a proximal locking device (PLD). Set screw was unscrewed by one-quarter of a turn after slightly tightening in all cases. Distal locking mode was based on the surgeon’s preferences, according to fracture’s stability and surgical technique. In Gamma3T nails, distal locking is indicated “to be used generally in dynamic mode, and in static mode only when the fracture is considered unstable, when the nail size does not fit the medullary canal size, or when there is a risk of interfragmentary rotation”. Distal screw did not fit distal hole in two cases. One was left as it was and was considered DN, the other case was correctly replaced as ST and was considered ST.

Pre-operative and peri-operative variables were obtained from clinical records, including serial blood tests and immediate pre-operative and post-operative radiological studies (anteroposterior projection of the pelvis and axial projection of the affected hip) as well as dependence and mobility using Fracture Mobility Score (FMS) [62]. Similar x-rays were obtained at each follow-up visit, if conducted. Partial weight bearing (PWB) with crutches or walker was allowed if a good reduction was considered by Fogagnolo criteria [24] immediately after surgery, or after first check-up. Post-discharge follow-up consisted of standard clinical–radiological check-up visits at first, third, fifth, ninth and twelfth month (± 1 week) after discharge, which concluded once the endpoint was reached. At each visit, they were evaluated with regard to pain at the fracture site, surgical wound and mobility using FMS [62]. No one was classified in the unknown item of the FMS scale, since according to the organisation chart (Fig. 1), patients who did not complete an endpoint were excluded. All complications were registered at the time they were diagnosed, as well as major complications that finally consolidated.

We have divided NTMC into two categories: major (may require surgical reintervention) and minor (no require surgical reintervention). By major, we understand cut-out, cut-in, cut-through, pull-out, breakage, osteonecrosis and pseudo-arthrosis, and by minor, we understand fracture collapse (also called caput–collum shortening or back-out) and loss of reduction. The last two can be combined. Significant fracture collapse was considered equal to or greater than 1 cm, as previously established by Zlowodzki [66] and Fang [21]. We have classified them as early mechanical complications (EMC) or late mechanical complications (LMC), depending on whether they occur within 6 months post-operatively or later, which is between the 3 months proposed by Bojan [7] and the first year indicated by Ehlinger [19].

Doppelt’s method [17] was used to detect non-obvious radiological complications and to measure tip–apex distance (TAD), given that diameter of cephalic screw is known (10.5 mm). Cleveland classification was used in immediate post-operative x-rays.

Other complications were recorded in clinical records. Infection met the confirmatory criteria to be assessed as an infection after fracture fixation (IAFF), as proposed by Metsemakers et al. [47]. Lateral thigh pain was considered when such pain was reported during follow-up, without association with new trauma or IAFF.

For the identification of the number of deaths during the follow-up period, we consulted the national death rate of the Spanish Ministry of Health at the end of February 2020 [to eliminate possible bias due to the emerging coronavirus disease 2019 (COVID-19) pandemic].

Regarding peri-operative variables, there are significant differences in all pre-operative classification systems (Jensen [29], AO/OTA until 2017 [42] and Massoud [44]), with ST being used more often in unstable fractures and DN in stable fractures. There are also significant differences in immediate post-operative loading, allowed more often in DN. There are no differences in TAD [4] or position according to Cleveland [14]. We also observed no differences in the post-operative Fogagnolo classification [24] (Table 1).

**Table 1:** Peri-operative variables

Peri-operative variables	Static distal locking (ST)	Dynamic distal locking (DN)	*p*
Number of cases	151	57	
Pre-Sx. classifications			
Jensen			0.002^a*^
I: simple, non-displaced	1 (0.7%)	4 (7.0%)	
II: simple or displaced basicervical	45 (29.8%)	27 (47.4%)	
III: displaced, extended to GT	10 (6.6%)	5 (8.8%)	
IV: displaced, extended to LT	67 (44.4%)	17 (29.8%)	
V: displaced, extended to GT/LT	28 (18.5%)	4 (7.0%)	
Stability (Jensen)			0.001^a*^
Stable (I + II)	46 (30.5%)	31 (54.3%)	
Unstable (III + IV + V)	105 (69.5%)	26 (45.6%)	
AO/OTA 2007			0.008^b*^
A1	39 (26.2%)	24 (50.0%)	
A2	93 (62.4%)	21 (43.8%)	
A3	17 (11.4%)	3 (6.3%)	
Stability (AO/OTA 2007)			0.000^b*^
Stable (A1 + A2.1)	84 (55.6%)	39 (68.4%)	
Unstable (A2.2 + A2.3 + A3)	65 (43.0%)	9 (15.8%)	
“Pure” basicervical (B2.1)	2 (1.3%)	9 (15.8%)	
Stability (Massoud)			0.007^b*^
Stable (Non-basicerv-equiv.)	10 (6.6%)	11 (19.3%)	
Unstable (Basicerv-equiv.)	141 (93.4%)	46 (80.7%)	
Medical			
Average stay (days)	9.71 (SD 3.48) (4;26)	9.54 (SD 2.96) (4;20)	0.985^c^
Pre-Sx. stay (days)	3.45 (SD 2.62) (0;9)	2.86 (SD 2.26) (0;7)	0.130^c^
Post-Sx. stay (days)	6.22 (SD 2.81) (3;25)	6.67 (SD 2.60) (3;20)	0.075^c^
Immediate post-Sx. PWB			0.003^b*^
No	90 (59.6%)	21 (36.8%)	
Yes	61 (40.4%)	36 (63.2%)	
Estimated blood loss (g/dl Hb)	1.816 (SD 1.79) (−3.5;5.8)	2.260 (SD 1.53) (−2.3;5.9)	0.140^c^
Total transfusions [Hem]	1.31 (SD 1.40) (0;8)	1.281 (SD 1.32) (0;5)	0.635^c^
Social situation			0.518^b^
At home alone	0 (0%)	0 (0%)	
At home accompanied	64 (42.4%)	27 (47.4%)	
Institutionalised	87 (57.6%)	30 (52.6%)	
Dependency			0.483^c^
Barthel	46.29 (SD 20.60) (10;80)	48.68 (SD 21.74) (10;85)	
Radiological			
TAD	22.958 (SD 6.50) (6.76;43.42)	22.42 (SD 6.67) (10.78;39.49)	0.647^c^
Cleveland quadrants (position) %			0.189^b^
Anterosuperior (1)	4 (2.6%)	0 (0%)	
Superior–central (2)	9 (6.0%)	0 (0%)	
Posterosuperior (3)	0 (0%)	0 (0%)	
Anterior–central (4)	15 (9.9%)	6 (10.5%)	
Centre–centre (5)	86 (57%)	32 (56.1%)	
Posterior–central (6)	8 (5.3%)	3 (5.3%)	
Anteroinferior (7)	2 (1.3%)	0 (0%)	
Inferior–central (8)	16 (10.6%)	6 (10.5%)	
Posteroinferior (9)	11 (7.3%)	10 (17.5%)	
C. Cleveland (centre versus no centre)			0.916^b^
Centre–centre (5)	86 (57.0%)	32 (56.1%)	
Rest of positions	65 (43.0%)	25 (43.9%)	
Post-Sx. classifications			
Fogagnolo			0.12^b^
Poor	9 (6.0%)	0 (0%)	
Acceptable	51 (33.8%)	17 (29.8%)	
Good	91 (60.3%)	40 (70.2%)	

Tests: ^a^ Fisher’s exact test. ^b^ Pearson’s chi-square test. ^c^ Mann–Whitney *U* test

GT, greater trochanter; LT, lesser trochanter; Sx., surgery; PWB, partial weight bearing

### Statistical analysis

Descriptive statistics generated using SPSS 20.0 (SPSS, Inc., Chicago, IL, USA) were utilised for data analysis. Kolmogorov–Smirnov tests were used to evaluate the Gaussian distributions of continuous variables, and comparisons were performed with Mann–Whitney *U* tests. For categorical variables, Pearson chi-square tests and Fisher’s exact tests were used, and for American Society of Anesthesiologists (ASA) risk score, median’s test was used. All *P*-values were two-sided, and *P*-values below 0.05 were considered significant. The Mantel–Cox log-rank test was used to evaluate survival.

## Results

Both groups are comparable in all pre-operative variables, non-modifiable (age, sex, fracture side) and modifiable (social situation, dependency according to Barthel’s index [40], comorbidity according to Charlson’s comorbidity score [11, 12], cognitive impairment according to Pffeifer’s classification [43, 55], severe osteoporosis by previous fractures [50], previous osteoporosis treatment, anti-platelet therapy/anti-coagulation therapy (APT/ACT) and ASA classification [22]) (Table 2).

**Table 2:** Pre-operative variables

Pre-operative variables	ST	DN	*P*
Number of cases	151	57	
Non-modifiable			
Average age (years)	85.50 (SD 6.87) (65;103)	85.96 (SD 6.65) (65;96)	0.359^c^
Sex (male/female) %			0.883^b^
Female	118 (78.1%)	44 (77.2%)	
Male	33 (21.9%)	13 (22.8%)	
Side			0.621^b^
Left	64 (42.4%)	22 (38.6%)	
Right	87 (57.6%)	35 (61.4%)	
Modifiable			
Social situation			0.886^b^
At home alone	22 (14.6%)	7 (12.3%)	
At home accompanied	80 (53.0%)	30 (52.6%)	
Institutionalised	49 (32.5%)	20 (35.1%)	
Dependency			
Barthel’s index	74.47 (SD 22.24) (15;100)	76.41 (SD 19.63) (10;100)	0.823^c^
Comorbidity			
Charlson Comorbidity Index (not age-adjusted)	2.12 (SD 1.36) (0;7)	2.25 (SD 1.04) (0;4)	0.209^c^
Charlson Comorbidity Index (age-adjusted)	6.09 (SD 1.37) (4;11)	6.17 (SD 1.05) (4;8)	0.277^c^
Cognitive impairment (Pfeiffer’s)			0.306^b^
None	28 (18.5%)	8 (14.0%)	
Mild	97 (64.2%)	40 (70.2%)	
Moderate	26 (17.2%)	8 (14.0%)	
Severe	0 (0%)	1 (1.8%)	
Osteoporosis (previous fractures, Nuti’s definition)			0.769^b^
None	85 (56.3%)	36 (63.2%)	
Traumatic (non-osteoporotic)	5 (3.3%)	1 (1.8%)	
Osteoporotic:			
Hip	12 (7.9%)	3 (5.3%)	
Other locations	38 (28.6%)	6 (33.3%)	
Both	5 (3.0%)	3 (5.3%)	
Previous osteoporosis treatment*			0.596^b^
No	129 (85.4%)	47 (82.5%)	
Yes	22 (14.6%)	10 (17.5%)	
APT/ACT			0.06^b^
None	87 (57.6%)	32 (56.1%)	
Acetylsalicylic acid 100 mg	23 (15.2%)	19 (31.6%)	
Acetylsalicylic acid 300 mg	16 (10.6%)	1 (1.8%)	
Clopidogrel	1 (0.7%)	0 (0%)	
Acenocumarol	21 (13.9%)	5 (8.8%)	
Direct Xa inhibitors	2 (1.3%)	1 (2.2%)	
LMWH	1 (0.7%)	1 (1.8%)	
ASA Risk Score	3 (1;4)	2 (1;4)	0.893^d^

Tests: ^a^ Fisher’s exact test. ^b^ Pearson’s chi-square test. ^c^ Mann–Whitney *U* test. ^d^ Median’s test

APT, anti-platelet therapy; ACT, anti-coagulation therapy; LMWH, low molecular weight heparin; ASA, American Society of Anesthesiologists

*Upon admission, no calcium and/or vitamin D

### Consolidation

Consolidation is the most common in both locking types, with percentages higher than 95%. There is a mild, non-statistically significant trend towards longer time in the ST group versus the DN group (6.70 versus 6.07, *P* = 0.069).

### NTMC

Less than 6% of cases had major radiological complications. The differences between ST and DN were not significant. Cut-out occurred in most cases (8 out of 11; 50% in ST and 100% in DN). One of them (ST) was considered as late. Out of the seven early cut-out cases, four were considered complete and three incomplete. There were three cases associated with infection after fracture fixation (IAAF). All early cases occurred in ST nailing (four cases) or non-dynamised DN nailing (three cases). One late cut-through, one osteonecrosis and one pseudo-arthrosis (with an underlying coagulopathy) were also observed. Excluding late cases, average detection time was 5.8 weeks (ST) and 4.5 weeks (DN, with only three cases) (*P* = 0.699).

Great discrepancy was observed in minor complications, statistically significant in favour of ST (56.3% versus 36.8%, *P* = 0.028). The most frequent complication was clearly > 1 cm shortening (67 in ST, 78.8%, versus 11 in DN, 52.4%), followed by associated loss of reduction, and isolated loss of reduction. Once the three late cases (2.83%) were excluded, average detection time was 5.5 weeks (5.62 in ST versus 5.42 in DN, *P* = 0.815).

In total, radiological complications were statistically more frequent in ST than in DN (61.6% versus 42.1%, *P* = 0.012), although generally speaking there were no differences regarding the time when they occur (5.63 ST versus 5.34 DN, *P* = 0.938).

### Infection (IAFF)

Ten cases of IAFF were reported (4.8% in total), eight ST and two DN, with no differences between groups or in terms of diagnostic time (4.12 in ST versus 5.36 in DN, *P* = 0.188).

### Lateral thigh pain

After excluding the cases of infection, 11 cases of lateral thigh pain (5.5% in both groups) were also recorded during follow-up, with no significant differences. Their time of onset was highly variable, from a few weeks to more than 2 years. Eight cases occurred in ST settings, and three in DN cases that were not dynamised.

### Dynamisation

Only 21.1% of the DN cases involved dynamisation, with 5.42 weeks on average and for no longer than 8 weeks (Table 3).

**Table 3:** Consolidation and post-operative complications detected

Variable	ST	DN	*P*
Number of cases	151	57	
Consolidation^1^			
Number of cases (%)	147 (97.4%)	55 (96.5%)	0.667^a^
Weeks (average)^1^	6.70 (SD 3.71) (3;33)	6.07 (SD 2.69) (3;21)	0.069^c^
NTMC			
Minor			
Number of cases (%)	85 (56.3%)	21 (36.8%)	0.028^a*^
Loss of reduction (%)	4 (4.7%)	3 (14.3%)	
Fracture collapse > 1 cm	67 (78.8%)	11 (52.4%)	
Both	14 (16.5%)	7 (33.3%)	
Weeks (average)^2^	5.62 (SD 2.27) (0.86;17.14)	5.42 (SD 1.02) (3.86;8)	0.815^c^
Major			
Number of cases (%)	8 (5.29%)	3 (5.26%)	1.0^a^
Early cut-out (%)	4 (50%)	3 (100%)	
Late cut-out (%)	1 (12.5%)	0 (0%)	
Cut-in (%)	0 (0%)	0 (0%)	
Cut-through (%)	1 (12.5%)	0 (0%)	
Breakage (%)	0 (0%)	0 (0%)	
Pull-out (%)	0 (0%)	0 (0%)	
Osteonecrosis (%)	1 (12.5%)	0 (0%)	
Pseudo-arthrosis (%)	1 (12.5%)	0 (0%)	
Weeks (average)^3^	5.8 (SD 4.23) (2.14;12.86)	4.5 (SD .30) (4.29;4.71)	0.699^c^
Total			
Number of cases (%)	93 (61,6%)	24 (42.1%)	0.012^a*^
Weeks (average)^2,3^	5.63 (SD 2.39) (0.86;17.14)	5.34 (SD 1.02) (3.86;8)	0.938^c^
Dynamisation			
Number of cases (%)		12 (21.1%)	-
Weeks (average)		5.42 (SD 2.06) (1;8)	-
Other complications			
IAFF			
Number of cases (%)	8 (5.3%)	2 (3.5%)	0.731^a^
Weeks (average)	4.12 (SD 10.23) (0.29;29.43)	5.36 (SD 6.57) (0.71;10)	0.188^c^
Lateral thigh pain			
Number of cases (%)^4^	8 (5.5%)	3 (5.5%)	1.0^a^
Weeks (average)^4^	30.01 (SD 43.25) (2.85;117.14)	27.14 (SD 22.69) (7.86;52.14)	0.414^c^

Tests: ^a^ Fisher’s exact test. ^c^ Mann–Whitney *U* test

^1^ Excluding major complications preventing consolidation (five cases of early cut-out and one case of pseudo-arthrosis)

^2^ Excluding “late” diagnoses (three cases)

^3^ Excluding “late” diagnoses (four cases: one late cut-out; one late cut-through; one osteonecrosis and one pseudo-arthrosis)

^4^ Excluding new trauma or IAAF (ten cases)

### Mobility

**Fig. 2:**
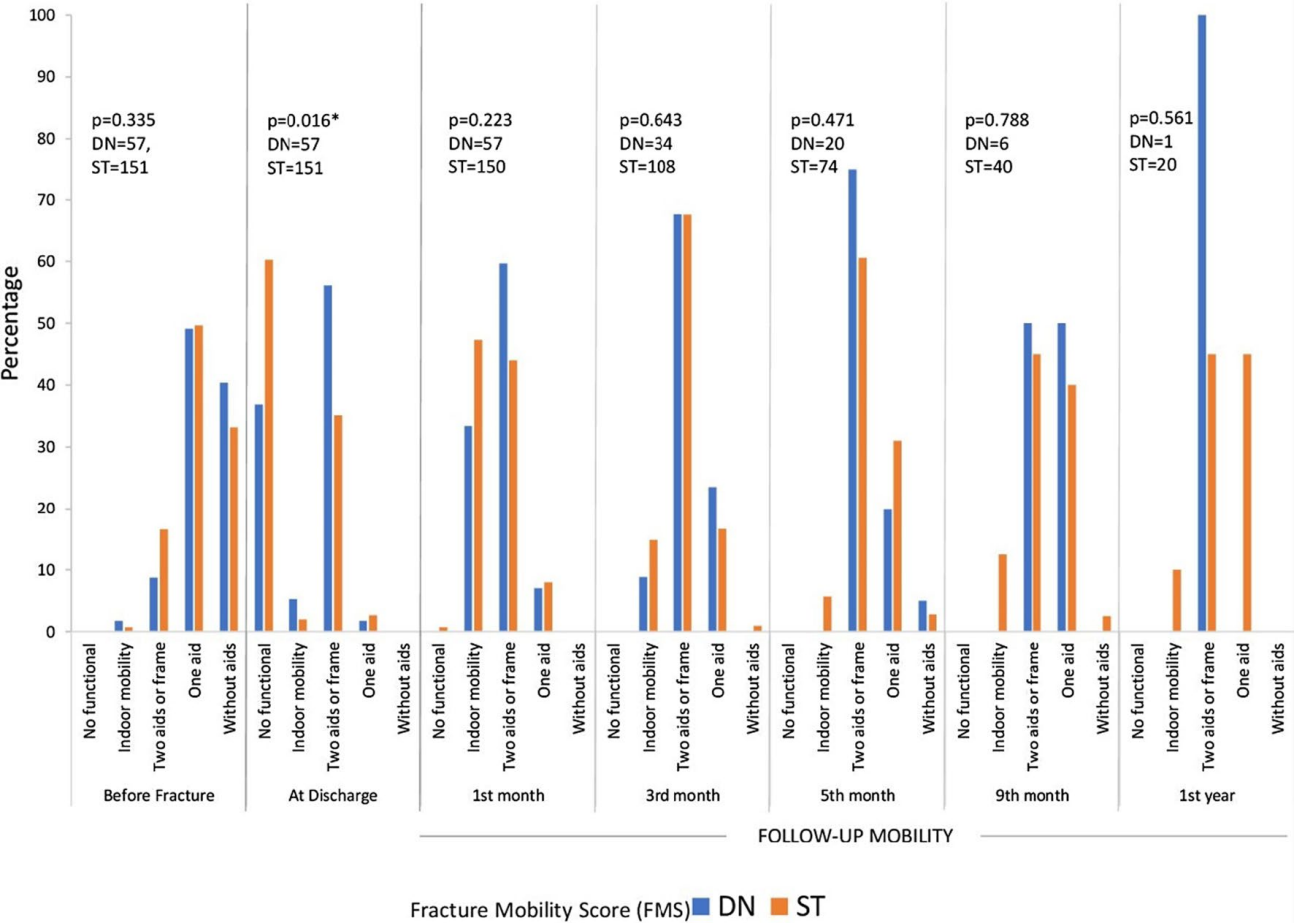
Patients’ mobility (Fisher’s exact test). FMS, Fracture Mobility Score

Statistically significant differences were detected only at the moment of discharge; fewer patients needed two aids per frame in DN. However, during standardised follow-up, there were no statistically significant differences (Fig. 2).

### Long-term survival

**Fig. 3:**
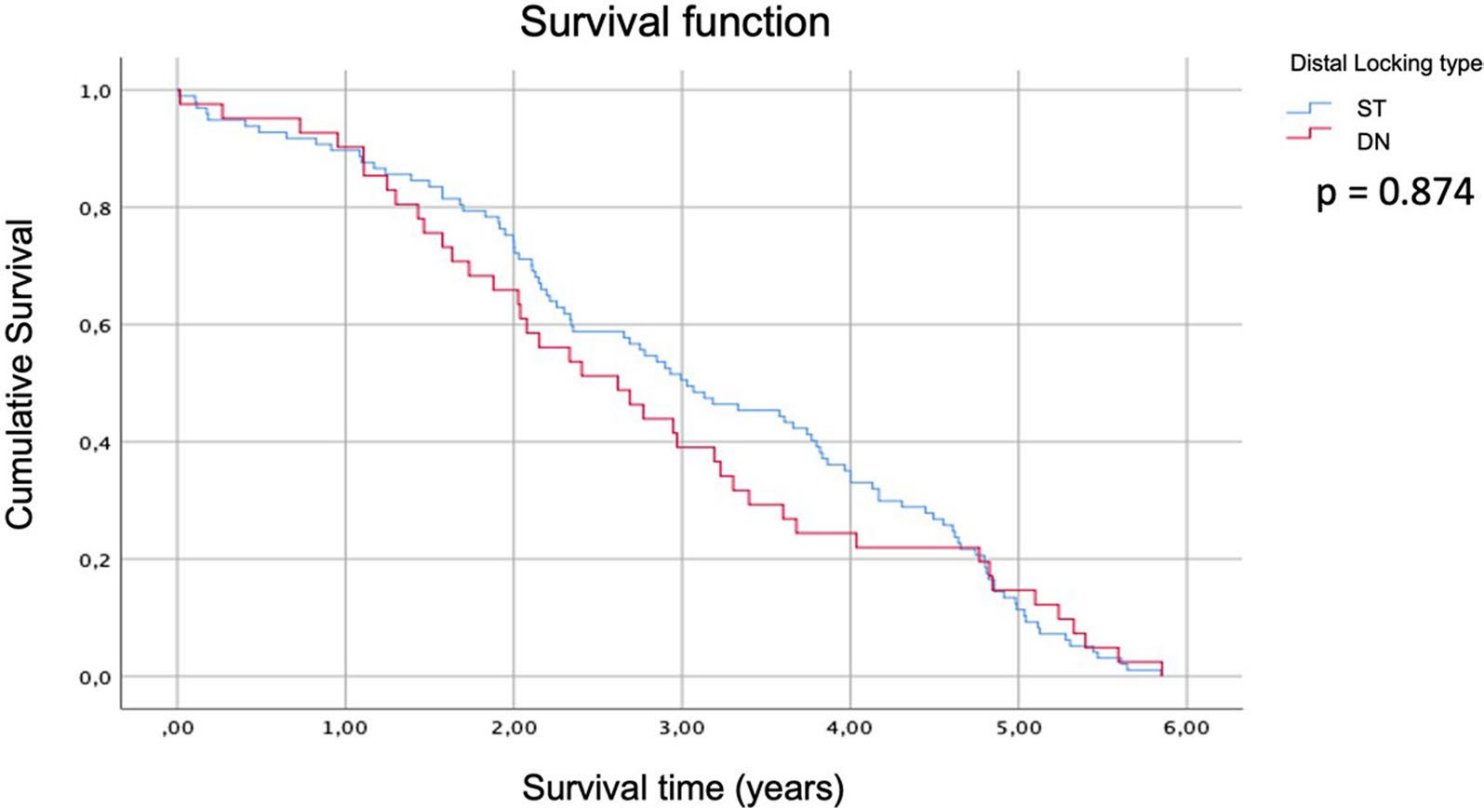
Patients’ survival

Long-term survival is similar in both distal locking types (*P* = 0.874), after almost 6 years follow-up (Fig. 3).

## Discussion

Controversy regarding distal locking in short nailing continues, mainly concerning “when and how”. Ozkan et al. [51, 52] do not approve of it in stable fractures, given shorter time, less surgical bleeding, and fewer intra-operative complications. On the contrary, its supporters stress lower bell-clapper effect, higher rotation stability, and latest designs reducing intra-operative and post-operative complications [39].

Distal locking is indicated in all unstable fractures. However, to date, classifications have no prognostic value, perhaps because they do not evaluate intra-operative and post-operative stability. Evans [20] suggested the importance of the internal cortical layer to transform an unstable fracture into a stable one, but Jensen determined the main fragments [29]. AO/OTA 2007 classification [42], used mainly for research purposes, has a high interobserver variability [54]. A new version (2018) has been developed with standardised projections and marking the role of the lateral wall, but it has scarce practical application. It maintains pure basicervical fractures, but independently of transcervical ones (from 31B2.1 to 31B3) [45]. Massoud considered AO/OTA 2007 A1.1 and all A2 types as “basicervical-equivalent”, because their disrotation tendency [44].

Our usual practice shows a clear trend of DN locking significantly more often in stable patterns belonging to the three classifications mentioned, and of ST locking in unstable patterns. However, most of the few cases considered as “pure” basicervical were DN, probably due to traditionally considering as benign a pure pattern in two fragments. Use of distal locking in short nails is standard practice in unstable 31A3 or reverse obliquity fractures, and in those whose subtrochanteric extension indicates it [9]. Influence of position is only a theoretically higher rigidity of “in vitro” ST implants [35]. In our hospital, the use of distal locking is practically systematic (98.84%), considering all osteoporosis cases, independent of the pattern or the stability of the fracture.

Consolidation of ECF with nails is estimated in about 95% of cases [2, 5]; the rest are described as complications. However, there are some major mechanical complications that do not prevent consolidation. By applying these principles, our results have been better than the previous ones.

Description and naming of complications is still unclear and not universally accepted. Instrumental improvements have reduced and changed their spectrum, as described by Bojan [6] and Ehlinger [19].

Cut-out is the paradigm of major complications, mostly those based on cephalic screws [28]. Out of the 11 major complications, 8 are cut-out cases. Initial literature review suggested that the incidence of cut-out was up to 12.6% with the Richard’s sliding hip screw [15]. However, it has decreased to less than 8% from 2004, and it is currently estimated to be 1.6–4.3% [3]. This study concluded an incidence of 3.84% in the ST and 5.26% in the DN. Minimising cut-out dates back to a long time ago [19]. A better implant design [46], and improvement in the learning curve [36] and in the placement of the cephalic device, essential after Baumgaertner’s work [4], may explain the results. From the latter, it is accepted that tip–apex distance (TAD) < 25 mm is a protection factor, with inter-observer reliability [31]. The rest of them have not proved to be so reliable. The distal tip in the centre–centre position seems to be protective too (Cleveland and Bosworth [14]), with no consensus on peripheral positions. These factors are similar in both types, as well as Fogagnolo’s classification [24]. Seven out of the eight were early. Bojan [7] suggests that there could be an underlying biological problem in late ones. Although we excluded confirmed pathological fractures, in our late cut-out case local vascularisation could have been compromised by two cerclage wires. Like others [6], we consider that cut-out is the result of unfavourable biological and insufficient mechanical conditions (reduction quality, osteosynthesis accuracy and post-operative stability). All early cut-out cases occurred in potentially more rigid systems (ST and non-dynamised DN). Neither have we observed other major complications such as cut-in [65] or pull-out [58], which are relatively frequent (5–8%) in cephalic helical blade devices. With hardly any case reports on the former [56] and almost no references of pull-out, the latest would be related to surgical technique errors [23] because design and tightening of the set screw prevent it. Theoretic biomechanical helical blade superiority is harmed by its migration “in vivo” due to the lack of set screw, an aspect that was attempted to be corrected in subsequent developments [33]. Nail breakage, which is currently an exceptional complication, was not observed either [25]. There are no differences in major complications, probably due to the scarce incidence recorded.

More minor complications were detected in ST. Most relevant is fracture collapse, linked to Trendelenburg gait and already reported with DHS [18] or PFN-A (Proximal Femoral Nail Anti-Rotation) in up to 15% of cases [60]. Protrusion of material into fascia lata and/or the loss of femoral offset due to back-out could explain this. It is unknown at what distance it starts being relevant, although 1 cm seems to have been accepted [21, 66]. This is why we chose said limit. The second-most relevant complication is loss of reduction associated with significant shortening and, finally, isolated loss of reduction. Minimising these could improve our patients’ gait, and therefore their quality of life.

Total number of complications is greater in ST than in DN, due to the higher relative proportion of minor ones. Subsequent studies must determine if unstable patterns or rigid fixation systems are decisive factors. Most studies deal with EMC [31], and we approve their importance, so average detection was around 5 weeks post-surgery.

Incidence of IAFF is very similar to previously reported [41] and clearly superior to the recent Norwegian series [27], although the latter involved re-interventions with short and long nails. IAFF could be considered as a possible risk factor for cut-out.

Reported lateral thigh pain is within previously observed ranges [16, 27]. It could be related to soft tissue irritation (iliotibial band) based on back-out or loss of reduction. All our cases are associated to ST or DN systems that were not dynamised, and in > 50% of cases they coincide with significant shortening.

Dynamisation was observed in less than a quarter of cases and always before 2 months. After the corresponding bibliographic review, we have not found any other work that reviews this aspect. It would be convenient, therefore, to go deeper into the reasons that give rise to this phenomenon.

This study did not allow immediate tolerated PWB for all, but we only detected statistically significant differences at the time of discharge, in favour of DN according to the FMS [62]. We emphasise that DN is associated with stable fracture patterns, and also surgical technique recommends ST in unstable ones. Distal locking does not seem to influence the final mobility, regardless of the time of started effective loading. Most of the current studies allow immediate partial or total weight bearing at discharge [61, 64], based on higher theoretic loads while lying down [49], but despite systemic benefits for the patient, its influence on NTMC is unclear [38]. On the other hand, many of them also do not indicate at what time the load allowed was effective, the protocol used, or its duration, with great uncertainty in this topic [37].

There is no previous literature about long-term security in distal locking. As expected, its type does not influence the overall survival over 5 years.

Until present, this is the first study which implemented a systematic follow-up until endpoint using the same nail. The majority of confound factors were controlled, including age, sex, comorbidity, main pre-operative classifications, weight bearing and mobility, but until now we do not have a useful prognosis classification. We did not find differences in consolidation, but we found important differences in NTMC, which was notably lower in DN. As static locking may present more minor complications, we could rethink their use. Thus, ECF with adequate stability and contact after implant, in which significant caput–collum shortening is not expected, could benefit from static distal locking. On the contrary, in unstable ECF after osteosynthesis, a theoretical less rigid set-up with dynamic distal locking maybe would be more desirable, with potential for controlled fracture collapse. These findings contradict previously described surgical technique.

A small percentage of distal dynamic locks dynamised, and all occurred relatively early, an aspect that will need to be explored further. As suspected, infection does not seem to be influenced, but it is interesting that lateral thigh pain cases occur in the stiffer assemblies. Long-term survival is the same, implying safety in both types.

Our retrospective non-randomised design implies some peri-operative differences, as well as the exclusion of UL cases. However, we have not found any previous research which studied the long-term outcomes. Thirty-seven (17.7%) patients did not reach the endpoint. Most were deaths, an expected percentage due to the characteristics of the sample.

## Conclusions

Any type of distal locking seems to be safe with Gamma3T. Consolidation was higher than 95% at 3 months in both groups, even sometimes when major complications were present. All NTMC were usually early (EMC) and occurred between the fifth and sixth weeks. Cut-out was the most frequent major complication, whereas other complications typically from helical blades systems were exceptional, which is a crucial advantage. ST tends to be related to more minor mechanical complications, especially significant back-out. There were no differences in IAFF but this may be related to cut-out, and lateral thigh pain can be related with fracture collapse. Less than 25% of the DN were indeed dynamised, and never in more than 2 months. Mobility was better at discharge only in DN ones, probably because of the theoretic higher stability perceived by surgeons. Long-term survival was similar.

## Data Availability

Datasets generated and analysed during the current study are not publicly available because data are not public, but they are available from the first author on reasonable request.

## Abbreviations

ECF: Extracapsular fracture
DHS: Dynamic Hip Screw
AO/OTA: AO Foundation/Orthopaedic Trauma Association
NTMC: Non-traumatic mechanical complications
ST: Static locking mode
DN: Dynamic locking mode
UL: Unlocked mode
PLD: Proximal locking device
FMS: Fracture Mobility Score
PWB: Partial weight bearing
EMC: Early mechanical complications
LMC: Late mechanical complications
TAD: Tip–apex distance
IAFF: Infection after fracture fixation
ASA: American Society of Anesthesiologists
APT: Anti-platelet therapy
ACT: Anti-coagulant therapy
PFN-A: Proximal Femoral Nail Anti-Rotation
